# Extracellular Fibrinogen-binding Protein (Efb) from *Staphylococcus aureus* Inhibits the Formation of Platelet-Leukocyte Complexes*[Fn FN2]

**DOI:** 10.1074/jbc.M115.678359

**Published:** 2015-12-01

**Authors:** Mareike G. Posner, Abhishek Upadhyay, Aisha Alsheikh Abubaker, Tiago M. Fortunato, Dina Vara, Ilaria Canobbio, Stefan Bagby, Giordano Pula

**Affiliations:** From the Departments of ‡ Biology and Biochemistry and; § Pharmacy and Pharmacology, University of Bath, Bath BA2 7AY, United Kingdom and; the ¶ Department of Biology and Biotechnology, University of Pavia, 27100 Pavia PV, Italy

**Keywords:** bacterial pathogenesis, fibrinogen, monocyte, neutrophil, platelet, proteomics, Staphylococcus aureus (S. aureus), P-selectin, PSGL-1, protein-protein interactions

## Abstract

Extracellular fibrinogen-binding protein (Efb) from *Staphylococcus aureus* inhibits platelet activation, although its mechanism of action has not been established. In this study, we discovered that the N-terminal region of Efb (Efb-N) promotes platelet binding of fibrinogen and that Efb-N binding to platelets proceeds via two independent mechanisms: fibrinogen-mediated and fibrinogen-independent. By proteomic analysis of Efb-interacting proteins within platelets and confirmation by pulldown assays followed by immunoblotting, we identified P-selectin and multimerin-1 as novel Efb interaction partners. The interaction of both P-selectin and multimerin-1 with Efb is independent of fibrinogen. We focused on Efb interaction with P-selectin. Excess of P-selectin extracellular domain significantly impaired Efb binding by activated platelets, suggesting that P-selectin is the main receptor for Efb on the surface of activated platelets. Efb-N interaction with P-selectin inhibited P-selectin binding to its physiological ligand, P-selectin glycoprotein ligand-1 (PSGL-1), both in cell lysates and in cell-free assays. Because of the importance of P-selectin-PSGL-1 binding in the interaction between platelets and leukocytes, we tested human whole blood and found that Efb abolishes the formation of platelet-monocyte and platelet-granulocyte complexes. In summary, we present evidence that in addition to its documented antithrombotic activity, Efb can play an immunoregulatory role via inhibition of P-selectin-PSGL-1-dependent formation of platelet-leukocyte complexes.

## Introduction

*Staphylococcus aureus* is responsible for numerous ailments in both humans and animals, ranging from superficial infections to more serious invasive diseases in which *S. aureus* enters the circulation and establishes infection foci in internal tissues, for example in the heart and bone marrow, leading to endocarditis and osteomyelitis, respectively (1). This invasive capability of *S. aureus* is conferred by bacterial adhesins that bind specifically to components of the host's plasma and extracellular matrix, such as fibrinogen and fibronectin (2). *S. aureus* also produces and secretes numerous proteins that aid infection and counter the host immune response, including coagulase (Coa), extracellular matrix-binding protein (Emp), extracellular adhesive protein (Eap), staphylococcal complement inhibitor (SCIN), Sbi, chemotaxis inhibitory protein (CHIPS), and extracellular fibrinogen-binding protein (Efb)^3^ (3–5). Efb is a 165-amino acid extracellular protein that binds fibrinogen (6) and platelets (7). Efb comprises a 29-residue N-terminal secretion signal followed by a disordered N-terminal half (Efb-N, residues 30–104) with two repeated regions (residues 46–67 and 77–98) that are homologous to the five to eight repeats in *S. aureus* coagulase (6) and a tri-helical bundle C-terminal domain (Efb-C, residues 105–165) (8). Efb was originally reported to have two binding sites for fibrinogen, one located in Efb-N (9) and the other located in Efb-C (10). Subsequent studies, however, found that Efb-C is not involved in binding fibrinogen (11, 12) and that there are two fibrinogen binding sites within Efb-N, each including one of the aforementioned coagulase-like repeats (11). On the other hand, Efb-C seems to play an immunosuppressive role by interfering with the complement system (8, 13). Efb and its homologue Ehp (Efb homologous protein, also known as Ecb for extracellular complement-binding protein) are essential for *S. aureus* virulence *in vivo* (14). In fact, *S. aureus* infections are significantly exacerbated *in vivo* in the presence of Efb (15, 16), and Efb impairs wound healing (17). Interestingly, Efb has previously been shown to inhibit platelet aggregation *in vitro* (18) and hemostasis *in vivo* (19). The inhibition of platelet activation and thrombus formation by Efb has been suggested to facilitate *S. aureus* survival in the blood and to aggravate *S. aureus* infection (20). Despite these important observations, the Efb mechanism of action has not yet been fully characterized. Efb antiplatelet activity has been suggested to depend on the inhibition of platelet-fibrinogen binding by Efb-N (21), but solid experimental confirmation of this hypothesis remains elusive.

In this study, we first assessed which part of Efb is responsible for Efb antithrombotic activity using recombinant Efb, Efb-N, and Efb-C constructs. We then analyzed the effect of these constructs on fibrinogen binding of resting and activated platelets and determined the platelet binding ability of the Efb constructs. We then identified novel Efb-binding platelet proteins. One such protein, P-selectin, which binds Efb directly in the absence of fibrinogen, mediates platelet-leukocyte complex formation via its interaction with PSGL-1 (22, 23). We have shown that Efb inhibits both P-selectin-PSGL-1 binding and platelet-leukocyte complex formation. Other bacterial proteins, such as *S. aureus* staphylococcal superantigen-like 5 (SSL5) and staphylococcal enterotoxin-like X (SEIX), have been shown to bind PSGL-1 on neutrophils and interfere with the interaction between PSGL-1 and P-selectin (24, 25).

In summary, we have characterized a novel mechanism of action of Efb as an inhibitor of platelet-leukocyte complexation via abolition of P-selectin-PSGL-1 interaction. Because platelet-leukocyte complexes play an increasingly recognized role in the innate immune response (26, 27), this study provides an additional explanation for the immunosuppressive activity of Efb.

## Experimental Procedures

### 

#### 

##### Expression of Efb Protein Constructs

Recombinant full-length Efb (residues 29–165, cloned in pET15b), Efb-N (29–103, pBG100), and Efb-C (101–165, pSV281) were expressed in BL21(DE3) (Efb and Efb-N) and Arctic Express (DE3)RIL (Efb-C). The plasmids pBG100 and pSV281 were kindly provided by the Vanderbilt University Center for Structural Biology. Expression of Efb and Efb-C was induced overnight at 12 °C, and expression of Efb-N was induced for 1 h at 37 °C. Proteins were purified using Ni-affinity chromatography. Fractions containing pure protein were pooled, concentrated to 1 ml, and dialyzed into a modified Tyrode's-HEPES buffer (10 mm HEPES, 145 mm NaCl, 2.9 mm KCl, 1 mm MgCl_2_, 5 mm glucose, pH 7.3).

##### Whole Blood, Platelet-rich Plasma (PRP), and Washed Platelet Preparation

Human blood was drawn from healthy volunteers by median cubital vein venipuncture under local ethics committee approval. Sodium citrate was used as anticoagulant (0.5% w/v for platelet isolation, 0.1% w/v for whole blood experiments). PRP was separated from whole blood by centrifugation (200 × *g*, 15 min), and platelets were separated from PRP by a second centrifugation step (400 × *g*, 10 min), in the presence of prostaglandin E1 (PGE1, 40 ng/ml) and indomethacin (10 μm). Washed platelets were then resuspended in a modified Tyrode's-HEPES buffer (see above) at a density of 4 × 10^8^/ml.

##### Thrombus Formation

The Bioflux200 system (Fluxion, South San Francisco, CA) was used to analyze thrombus formation in human whole blood under flow (shear rate = 1000 s^−1^). Microchannels were coated with 0.1 mg/ml collagen I (monomeric collagen from calf skin). PRP was isolated from blood by centrifugation (200 × *g*, 15 min) and incubated for 1 h with Calcein^TM^ (5 μg/ml) at 37 °C to facilitate thrombus visualization in whole blood. Labeled platelets were then isolated from PRP (centrifugation), resuspended in unlabeled plasma, and added to the red blood cell fraction to reconstitute whole blood with physiological blood cell density (including platelets). Thrombus formation (on collagen) was then visualized by fluorescence microscopy. Representative pictures were taken at time 10 min, and surface area coverage was measured using the Bioflux200 Software (version 2.4), as described previously (28).

##### Fluorescent Labeling of Protein and Assessment of Labeled Protein Binding to Platelets by Flow Cytometry

Fibrinogen or purified recombinant Efb proteins were labeled with FITC, as described previously (29). Washed platelets (10^7^ ml^−1^) treated as indicated for the different experiments were labeled with FITC-fibrinogen (FITC-FGN, 50 μg/ml) or FITC-Efb proteins (1–3 μm). Platelet binding in the presence or absence of 3 mg/ml fibrinogen and 0.25 unit/ml thrombin was assessed by flow cytometry using a FACSAria III (BD Biosciences, Oxford, UK).

##### Proteomics, Pulldown Assays, and Immunoblotting

His-tagged Efb was bound to Sigma His-Select HF nickel affinity gel by co-incubation for 1 h at 4 °C with mild agitation. Platelet lysates were obtained by ultrasonication using a Branson 450 Digital Sonifier and incubated with the Efb-bound His-Select HF resin for 1 h at 4 °C with mild agitation, followed by washing four times with modified PBS (PBS with 300 mm NaCl and 20 mm imidazole added, pH 8). Efb-interacting platelet proteins were separated by running the His-Select HF resin on SDS-PAGE, and subsequently identified by mass spectrometry; peptides from tryptic digestion using a ProGest automated digestion unit (Digilab) were identified using a Dionex Ultimate 3000 nano-HPLC system in line with an LTQ-Orbitrap Velos mass spectrometer (Thermo Scientific). Four independent preparations were used. His-Select HF resin incubated with modified PBS, without Efb or Efb-N, was used as a negative control. Efb/Efb-N interactor proteins identified by mass spectrometry were confirmed by immunoblotting with specific antibodies and HRP-linked species-specific secondary antibodies using ECL detection. The antibodies used were: integrin α_IIb_β_3_ (Millipore AB1967), fibronectin and thrombospondin-1 (Thermo Scientific (MA5-12314 and MA5-13398, respectively), and multimerin-1 and PSGL-1 (Santa Cruz Biotechnology, sc-104427 and sc-13535, respectively). The P-selectin antibody used for immunoblotting was Bio-Vision 3633R-100, whereas the P-selectin antibody used for immunoprecipitation was Santa Cruz Biotechnology sc-6941. The inhibitory antibody for integrin α_IIb_β_3_ was BioLegend 359804.

##### P-selectin/Efb Interaction Measured by SPR

P-selectin-Fc (described under “P-selectin/PSGL-1 Co-immunoprecipitation”) was immobilized on SensiQ^TM^ COOHV sensor chips (SensiQ Technologies, Inc., Oklahoma City, OK) via amine coupling using 1-ethyl-3-(-3-dimethylaminopropyl) carbodiimide hydrochloride (EDC) and *N*-hydroxysuccinimide (NHS). SensiQ^TM^ COOHV sensor chips contain a high-capacity carboxymethyl dextran matrix. The SPR signal was recorded in the presence of different concentrations of full-length Efb (100 nm-2.8 μm). All experiments were performed using the SensiQ^TM^ Pioneer FE SPR platform (SensiQ Technologies). Experimental data were fit using the Qdat data analysis software. Curves were fitted using a least squares fitting algorithm.

##### P-selectin/PSGL-1 Co-immunoprecipitation

2 μg/ml recombinant P-selectin-Fc and PSGL-1-Fc (R&D Systems) were co-incubated for 2 h in PBS plus 1 mm CaCl_2_ for the cell-free assay or platelet lysates were obtained using immunoprecipitation lysis buffer (150 mm NaCl, 0.5% Nonidet P-40, 10 mm Tris, pH 7.3). The cell-free experiments were performed using P-selectin-Fc and PSGL-1-Fc (recombinant fusion proteins of P-selectin and PSGL-1 with immunoglobulin crystallizable fragments (Fc)) to allow efficient resuspension of P-selectin and PSGL-1. This is a common strategy to analyze membrane proteins that have low hydrophilicity (30, 31). Where indicated, 5 μm full-length Efb or Efb-N was incubated with the protein suspensions/platelet lysates. Either P-selectin or PSGL-1 (10 μg/ml) was immunoprecipitated using specific antibodies. Immunoprecipitated and co-immunoprecipitated proteins were identified by immunoblotting.

##### Whole Blood Flow Cytometry and Detection of Platelet-Leukocyte Complexes

Human whole blood samples were incubated with 5 μm full-length Efb or Efb-N for 30 min. Red blood cells were removed using Red Cell Lysis solution (Miltenyi Biotec), and the remaining cells were stained with anti-CD42b, anti-CD66, and anti-CD38 antibodies for the identification of platelet-, granulocyte-, and monocyte-specific events respectively. Flow cytometry was performed using a FACSAria III (BD Biosciences).

## Results

### 

#### 

##### Efb-N Binds Platelets and Exerts Antithrombotic Activity

**FIGURE 1.**
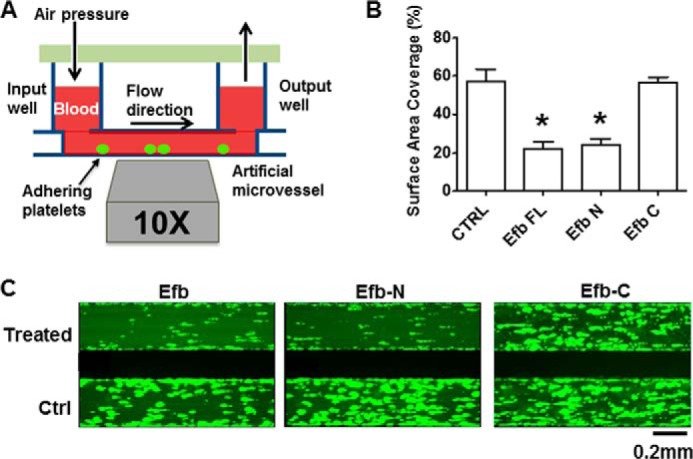
**Efb and Efb-N inhibit whole blood thrombus formation.** Platelets in human PRP were labeled by incubation for 1 h with 5 μm calcein AM before reconstitution of whole blood. Artificial microvessels were coated with 0.1 mg/ml collagen and infused with reconstituted blood at a shear rate of 1000 s^−1^. Thrombus formation was followed by fluorescence microscopy for 10 min, in the presence of 5 μm Efb, Efb-N, or Efb-C. In all cases, the control was a microvessel infused with whole blood with labeled platelet plus 5 μm BSA. *A*, the Bioflux200 apparatus. A multichannel lid applies pressure to an input well, which in turn feeds blood under physiological shear stress into a microvessel mounted on a fluorescence microscope. *B*, the results from five independent experiments were analyzed and are displayed as means ± S.E. The statistical significance of the difference was analyzed by one-way ANOVA with Bonferroni post test (* = *p* < 0.05, *n* = 5). Ctrl, control. *C*, representative examples of the results with aggregated platelets shown in *lighter green*.

A thrombus formation assay in whole human blood under physiological shear stress conditions is a powerful tool to analyze the hemostatic response and its pro-thrombotic aberrations *in vitro*. The antithrombotic activity of Efb has not been tested in this experimental model. We therefore used a previously described microfluidics assay (28, 32) to identify the domain of Efb that inhibits thrombus formation under these conditions. We used recombinant full-length Efb, Efb-N, and Efb-C. Both Efb and Efb-N strongly inhibited thrombus formation in whole human blood in physiological conditions, whereas Efb-C did not significantly affect thrombus formation (Fig. 1). The thrombus formation assay used here models the hydrodynamic conditions and intercellular interactions leading to thrombus formation in the bloodstream and confirms the previously suggested potential of Efb as an antithrombotic agent (18, 19). These data strongly suggest that Efb-N binds to platelets and exerts the antithrombotic activity of Efb.

##### Efb Binds to Platelets via Both Fibrinogen-dependent and Fibrinogen-independent Mechanisms

**FIGURE 2.**
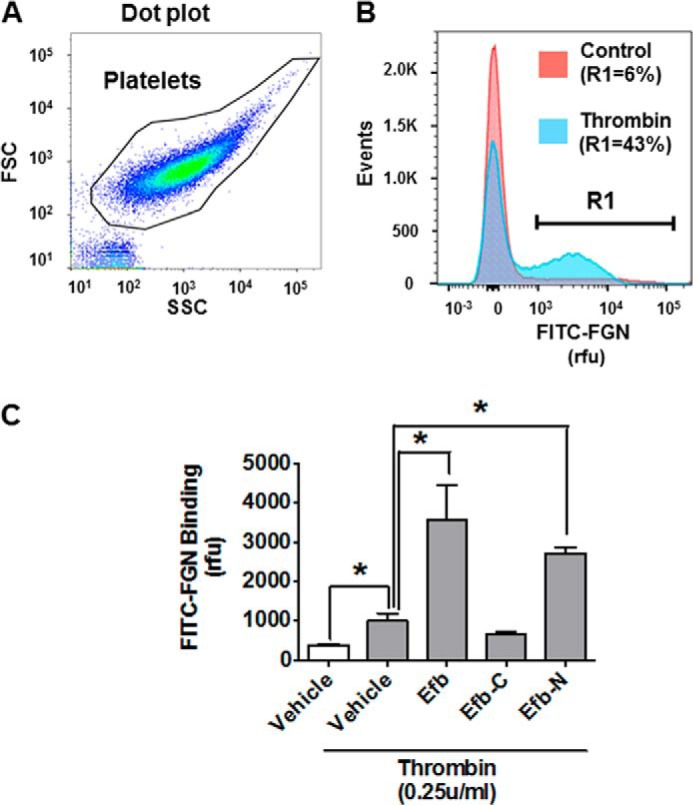
**Full-length Efb promotes fibrinogen binding by platelets.** Binding of 50 μg/ml FITC-fibrinogen by platelets (10^7^ ml^−1^) in resting conditions or treated with vehicle (5 μm BSA), 5 μm Efb, Efb-N, or Efb-C in the presence of 0.25 unit/ml thrombin was measured by flow cytometry using a FACSAria III (*n* = 3, * = *p* < 0.01 by ANOVA with Bonferroni post test). *A*, the forward scattering (*FSC*)/side scattering (*SSC*) dot plot of the platelet population. *B*, event histogram for FITC labeling of resting and thrombin-stimulated platelets. *R1* is the subpopulation of platelets with FITC labeling >1000 rfu, which represents 6% ± 5 and 43% ± 13% of the total population in resting and thrombin-stimulated platelets, respectively. *C*, the mean value for FITC-fibrinogen binding for the platelet population is presented as mean ± S.E. Statistical significance was tested by one-way ANOVA with Bonferroni post test (* = *p* < 0.01, *n* = 4).

Building on previous studies that did not provide a complete and definitive picture on the effect of Efb on fibrinogen binding by platelets and did not characterize the effect of Efb-N and Efb-C separately (11, 18, 19, 21), we investigated the effect of recombinant Efb, Efb-N, or Efb-C on washed platelet binding of fibrinogen. As expected, fibrinogen binding was minimal in resting conditions and was significantly increased by platelet stimulation by 0.25 unit/ml thrombin. Surprisingly, Efb and Efb-N mediated an increase rather than a decrease in fibrinogen binding upon platelet activation with thrombin (Fig. 2).

**FIGURE 3.**
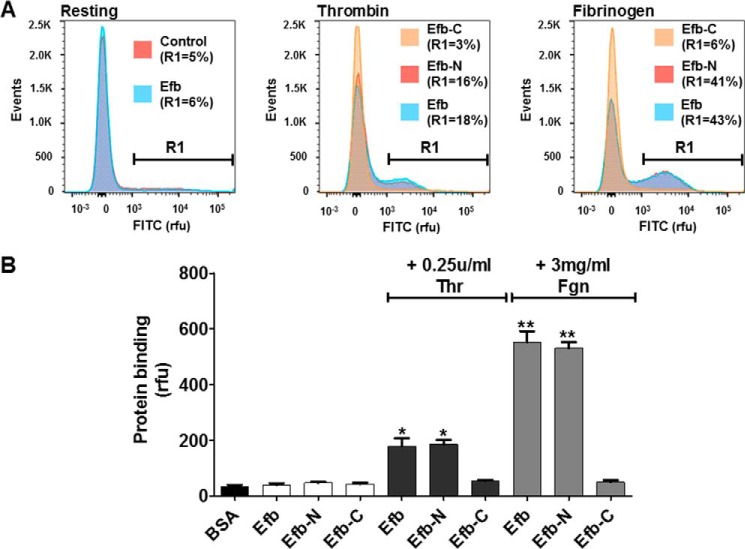
**Interaction of the N-terminal region of Efb with platelets.** FITC-labeled Efb, Efb-N, Efb-C or BSA (negative control) was incubated at 1 μm concentration with human platelets (10^7^ ml^−1^). Where indicated, the incubation was performed in the presence of 0.25 unit/ml thrombin (*Thr*) or 3 mg/ml FGN. Protein binding was assessed by flow cytometry using a FACSAria III. *A*, representative FITC-labeling histograms. *R1* is the subpopulation of platelets with FITC labeling >1000 rfu. *B*, average FITC labeling for different conditions is reported with S.E. Statistical significance was tested by one-way ANOVA with Bonferroni post test (* = *p* < 0.01 compared with BSA, ** = *p* < 0.01 compared with thrombin only for the same protein construct, *n* = 3).

We next assessed whether Efb can bind washed platelets directly. Efb, Efb-N, or Efb-C was FITC-labeled and used in flow cytometry experiments with washed platelets. Both Efb and Efb-N bound to washed platelets, but only in the presence of thrombin (0.25 unit/ml) or extracellular fibrinogen (3 mg/ml) (Fig. 3).

**FIGURE 4.**
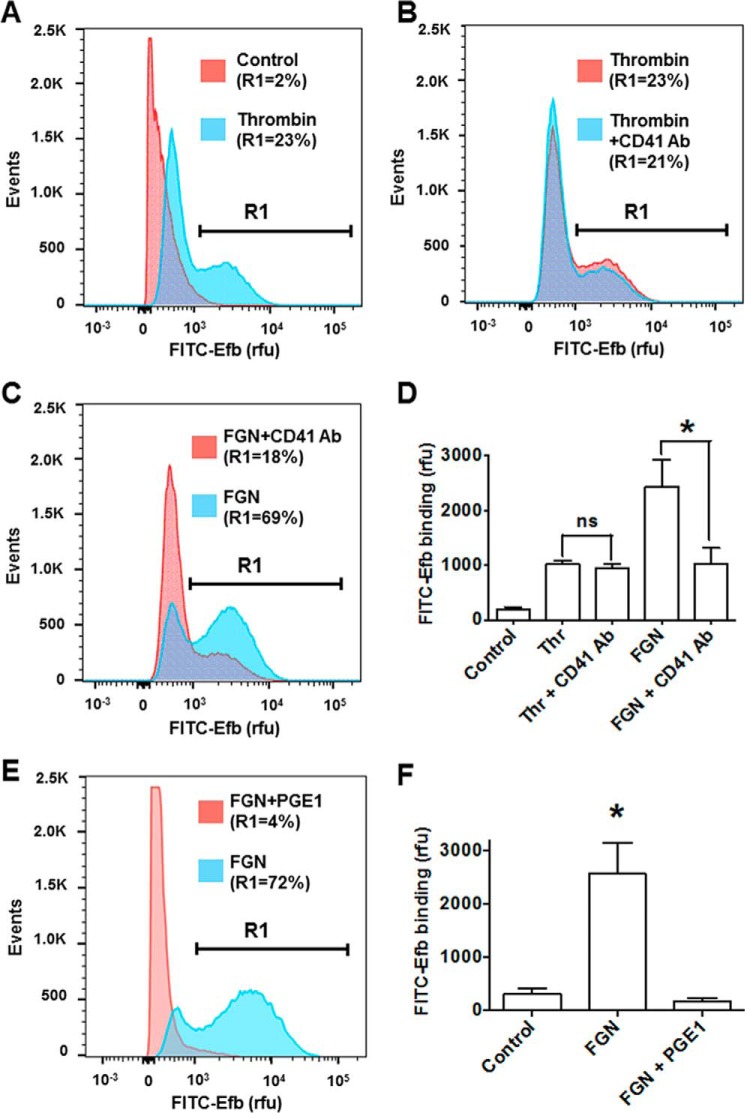
**Integrin α_IIb_β_3_ mediates the binding of Efb by platelets in the presence of fibrinogen.** FITC-labeled Efb was incubated at 3 μm concentration with human platelets (10^7^ ml^−1^), and binding was assessed by flow cytometry. Representative histograms are shown in *A*, *B*, *C*, and *E*. Where indicated, the incubation was performed in the presence of 50 μg/ml integrin α_IIb_β_3_ inhibitory anti-CD41 antibody (CD41 Ab) (*A–D*) or 40 ng/ml PGE1 (*E* and *F*). Mouse IgG1 κ isotype antibodies were used as negative control in *B* and *C*. The experiments were performed in the presence of 0.25 unit/ml thrombin (*Thr*) or 3 mg/ml FGN, as indicated in the figures, and performed three times. Protein binding was assessed by flow cytometry using a FACSAria III (*n* = 3). In *A*, *B*, *C*, and *E*, representative FITC-labeling histograms are shown with the percentage of events above 1000 rfu (indicated as *R1*). The data are quantified in *D* and *F*, where the mean fluorescence value ± S.E. from three independent experiments is reported. Statistical significance was tested by one-way ANOVA with Bonferroni post test (* = *p* < 0.01, *n* = 3). *ns* = non-significant in the pairwise comparison.

To understand the molecular mechanisms underlying thrombin- and fibrinogen-dependent binding of Efb to washed platelets and to assess the role of integrin α_IIb_β_3_, we examined FITC-Efb binding to platelets by flow cytometry in the presence of an inhibitory antibody for this integrin. The inhibitory antibody for integrin α_IIb_β_3_ only abolished fibrinogen-dependent binding of Efb, but did not alter the binding of Efb by thrombin-activated washed platelets (Fig. 4). This suggests that fibrinogen-mediated binding of Efb to platelets is integrin α_IIb_β_3_-dependent, whereas Efb binding in the presence of thrombin without exogenous fibrinogen proceeds via a different mechanism and is integrin α_IIb_β_3_-independent. Indirectly, this suggests that the levels of endogenous fibrinogen released by washed platelets upon thrombin stimulation are significantly lower than 3 mg/ml, the exogenous fibrinogen concentration used in our experiments and the average concentration in plasma (33); this is consistent with previous studies (34, 35) involving the fibrinogen concentration released by washed platelets *in vitro*. In summary, our data suggest a dual mechanism of binding of Efb by platelets: an extracellular fibrinogen/integrin α_IIb_β_3_-dependent mechanism, and a fibrinogen-independent mechanism mediated by at least one unidentified platelet surface receptor. In addition, the fibrinogen-dependent binding of Efb to platelets appeared to depend on basal levels of platelet activation in the washed platelet preparations, because it was completely abolished by treatment with PGE1 (Fig. 4, *E* and *F*).

##### Identification of Novel Efb-binding platelet Proteins

We therefore decided to identify platelet proteins that interact with Efb using a proteomics approach. Pulldown experiments were performed using platelet lysates obtained by ultrasonication and His-Select HF resin conjugated to full-length Efb in the presence of 3 mg/ml added fibrinogen. Captured proteins were identified by mass spectrometry (a full list of proteomics results is shown in supplemental Table 1). Platelet surface/extracellular proteins preferentially captured by Efb-loaded resin in at least three out of four samples were selected using a minimum threshold ratio of pulldown peptide number to negative control of 1.40 and are shown in Table 1. In addition to known interactors of Efb (*i.e.* fibrinogen, complement C3, and plasminogen) (6, 13, 16), this analysis revealed novel potential partners of Efb, including functionally relevant proteins such as P-selectin (a platelet plasma membrane protein) and multimerin-1, emilin-1, fibronectin, and thrombospondin-1 (soluble extracellular proteins able to interact with cell adhesion receptors on platelets).

**TABLE 1:** **Platelet proteins interacting with Efb identified by pulldown and mass spectrometry analysis** Sigma His-Select HF nickel affinity gel was used to capture His-tagged Efb-interacting proteins from four independent platelet lysates obtained by ultrasonication. Peptides from tryptic digestion using a ProGest automated digestion unit (Digilab) were identified using a Dionex Ultimate 3000 nano-HPLC system in line with an LTQ-Orbitrap Velos mass spectrometer (Thermo Scientific). Four independent preparations were used for the Efb pulldown, and two independent preparations were used as a control using HIS-Select beads without Efb (“empty beads”). The average number of peptide hits for the pulldown samples is reported in the first numerical column, whereas the average number of hits for the negative controls (empty beads) is shown in the second numerical column. The ratio between numbers of peptides in Efb pulldowns and control pulldowns is shown in the third column, which quantifies the robustness of the proteomic identification of these proteins as true interactors of Efb.

Protein name [locus]	Peptide hits (sample)	Peptide hits (control)	Difference
**Known interactors**			
Plasminogen [PLMN_HUMAN]	243	92	2.64
Complement C3 [CO3_HUMAN]	529	222	2.38
Fibrinogen gamma chain [FIBG_HUMAN]	205	146	1.40

**Novel potential interactors**			
Platelet membrane			
P-selectin [Q5R349_HUMAN]	60	27	2.22
Protein unc-13 homolog D [UN13D_HUMAN]	54	30	1.80

**Extracellular/adhesion**			
Emilin-1 [EMIL1_HUMAN]	62	21	2.95
Multimerin-1 [MMRN1_HUMAN]	77	27	2.85
Fibronectin [B7ZLF0_HUMAN]	133	52	2.56
Thrombospondin-1 [TSP1_HUMAN]	135	86	1.58

**FIGURE 5.**
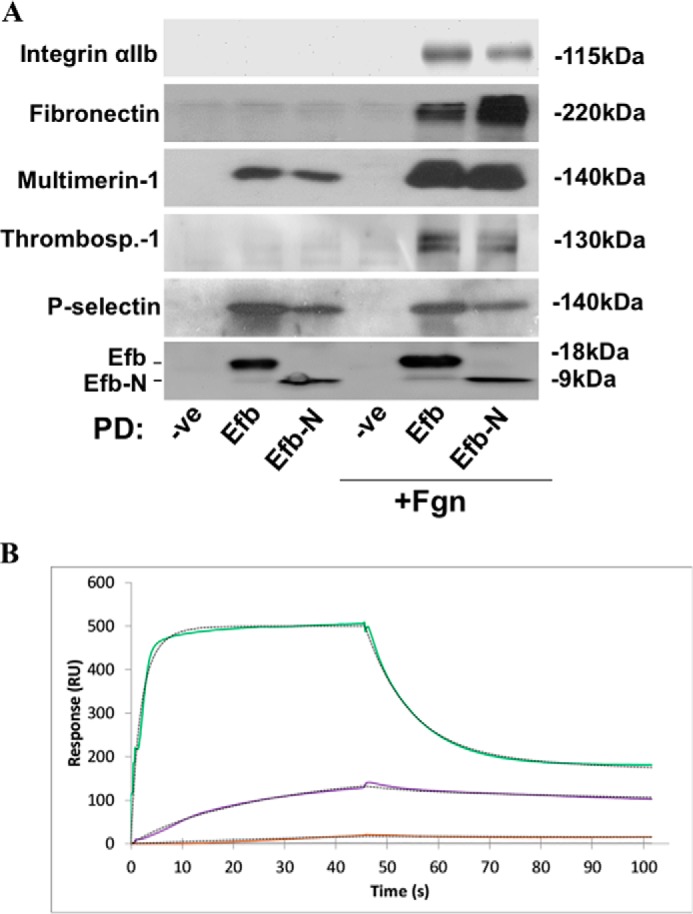
**Efb interacts with platelet P-selectin.** *A*, human platelets were ultrasonicated in the presence or absence of 3 mg/ml added fibrinogen. Sigma His-Select HF nickel affinity gel was used to capture His-tagged Efb- or Efb-N-interacting proteins from platelet lysates. Affinity precipitates were separated by SDS-PAGE and immunoblotted for integrin αIIb, P-selectin, multimerin-1, fibronectin, thrombospondin-1 (indicated by *Thrombosp.-1*), and His tag (to confirm Efb/Efb-N expression and immobilization). Immunoblots shown here are representative of those from three independent experiments. *-ve*, negative control; *PD*, pulldown. *B*, SPR data confirm the direct interaction of Efb and P-selectin. Recombinant P-selectin-Fc immobilized on COOHV-coated SensiQ^TM^ sensor chips was treated with increasing concentrations of Efb (100 nm (*brown*), 300 nm (*purple*), and 2.8 μm (*green*)). All experiments were performed using the SensiQ^TM^ Pioneer FE SPR platform (SensiQ Technologies). Experimental data were fit using the Qdat data analysis software. Curves were fitted using a least squares fitting algorithm. *RU*, response units.

Proteins identified by proteomics and characterized by expression on platelet surface or in human plasma were investigated further. Efb/Efb-N-interacting proteins were captured from platelet lysates (obtained by ultrasonication) using Efb/Efb-N-loaded His-Select HF resin and confirmed by immunoblot with specific antibodies (Fig. 5*A*). Integrin αIIb, fibronectin, multimerin-1, thrombospondin-1, and P-selectin were confirmed to interact with Efb and Efb-N in the presence of added extracellular fibrinogen. Interestingly, P-selectin appeared to bind Efb and Efb-N efficiently in the absence of added fibrinogen, whereas multimerin-1 showed some Efb binding activity without added fibrinogen, which was potentiated by adding fibrinogen. All other tested proteins depended on the addition of exogenous fibrinogen to bind Efb/Efb-N, suggesting that their interaction with Efb/Efb-N is fibrinogen-mediated rather than direct. The use of full-length Efb or Efb-N meant that these pulldown assays provided insight on the location of the interacting domain in Efb (the proteomics data set was obtained using full-length Efb only). For all tested proteins, the interaction with Efb-N was at least as effective as the interaction with full-length Efb, suggesting that Efb-N contains the binding site(s) for Efb interaction with its newly identified partners P-selectin, multimerin-1, fibronectin, and thrombospondin-1. Direct interaction of Efb with P-selectin was confirmed by SPR, revealing a *K_d_* for Efb-P-selectin interaction in the nm range (Fig. 5*B*).

**FIGURE 6.**
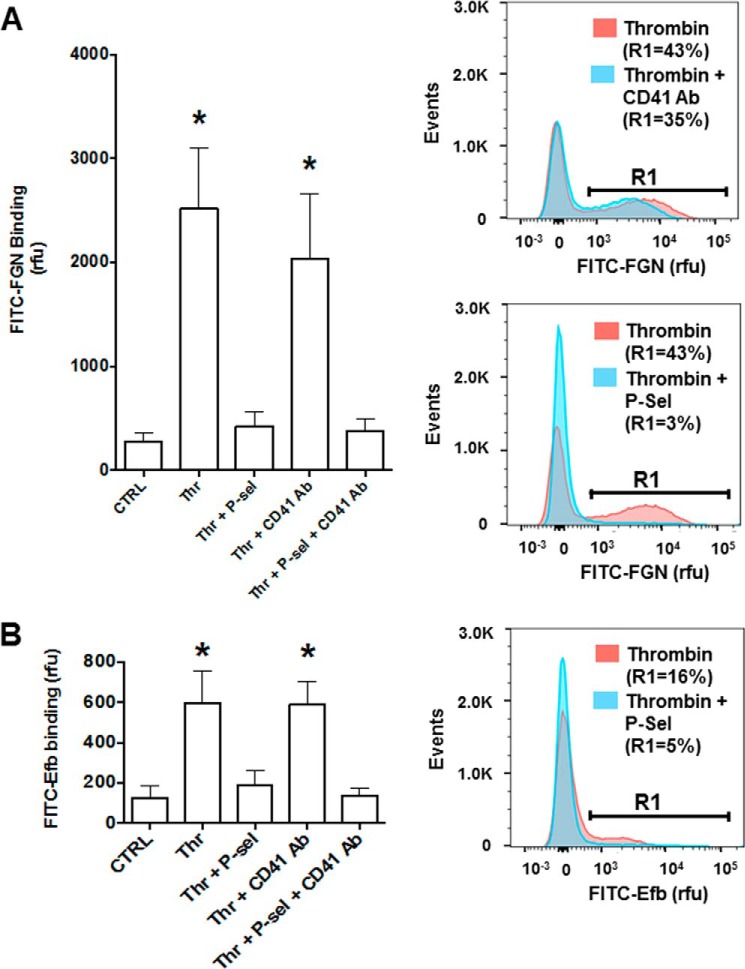
**P-selectin (*P-sel*) is responsible for Efb and fibrinogen binding by thrombin-stimulated platelets.** *A*, FITC-labeled FGN binding by platelets (10^7^ ml^−1^) in resting or thrombin- (*Thr*) stimulated (0.25 unit/ml) states was measured in the presence of 50 μg/ml P-selectin-Fc and/or 50 μg/ml integrin α_IIb_β_3_ inhibitory anti-CD41 antibody (CD41 Ab). *B*, FITC-labeled Efb was incubated at 1 μm concentration on human platelets (10^7^ platelets/ml) in resting (control (*CTRL*)) or thrombin-stimulated (0.25 units/ml) conditions. Where indicated, 50 μg/ml P-selectin-Fc and/or 50 μg/ml integrin α_IIb_β_3_ inhibitory anti-CD41 antibody (CD41 Ab) were added to the platelet suspension, and Efb binding was assessed by flow cytometry using a FACSAria III (*n* = 4). Representative FITC-labeling histograms are shown in the *right panels* with the percentage of events above 1000 rfu (indicated as *R1*). Mean fluorescence value ± S.E. from four independent experiments is reported in *left panels*. Statistical significance was tested by one-way ANOVA with Bonferroni post test (* = *p* < 0.01, *n* = 4).

Next, we tested whether P-selectin binding by Efb can participate in the increased binding of fibrinogen by thrombin-stimulated platelets shown in Fig. 2*C*. To obtain this information, we performed FITC-fibrinogen binding experiments in the presence of Efb and selective competitors of platelet P-selectin (*i.e.* soluble P-selectin-Fc fusion protein) and integrin α_IIb_β_3_ (*i.e.* inhibitory anti-CD41 antibody). These experiments showed that P-selectin is responsible for most of the binding of fibrinogen by activated platelets in the presence of Efb (Fig. 6*A*). We then confirmed that P-selectin mediates the interaction of Efb with platelets in the absence of fibrinogen by assessing the binding of FITC-Efb by thrombin-stimulated platelets in the presence of an excess of recombinant P-selectin-Fc (*i.e.* binding competition) (Fig. 6*B*). As shown above (Fig. 3), stimulation by thrombin is necessary to promote P-selectin-dependent binding of Efb by platelets. In addition, here we show that the presence of P-selectin-Fc fusion protein abolishes the binding of Efb by platelets. Taken together, these data suggest that the translocation and exposure of P-selectin on the platelet surface in response to thrombin (36) are necessary for Efb binding by platelets in the absence of exogenous fibrinogen.

##### Efb Disrupts Platelet-Leukocyte Interaction by Blocking P-selectin-PSGL-1 Interaction

**FIGURE 7.**
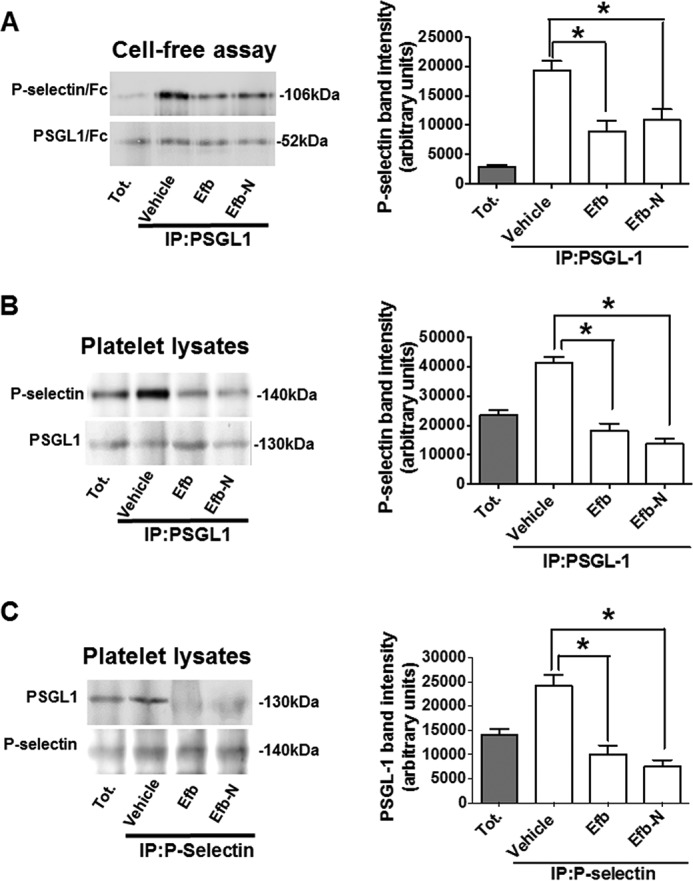
**Efb inhibits P-selectin-PSGL-1 interaction.** *A*, P-selectin-Fc (106 kDa) and PSGL-1-Fc (52 kDa) were mixed in PBS + 1 mm CaCl_2_ at a final concentration of 2 μg/ml and PSGL-1 was immunoprecipitated (*IP*) using 10 μg/ml anti-PSGL-1 antibody. *Tot.*, total. *B* and *C*, whole platelet lysates were immunoprecipitated using anti-PSGL-1 (*B*) or anti-P-selectin antibodies (*C*). Where indicated 5 μm Efb or Efb-N was added (equivalent to 45 and 90 μg/ml, respectively). In all cases, the immunoprecipitates were immunoblotted for P-selectin and PSGL-1. Immunoblots shown in this figure are representative of three independent experiments. The *right panels* represent the densitometric analysis of the immunoblots. Three independent experiments were analyzed with ImageJ for band mean intensity (minus background intensity), which is shown as average ± S.E. (arbitrary units). Statistical significance was assessed by one-way ANOVA with Bonferroni post test (*, *p* < 0.05, *n* = 3).

We subsequently focused on the biological role of the interaction between Efb and P-selectin. Co-immunoprecipitation experiments were conducted using recombinant P-selectin-Fc and PSGL-1-Fc (Fig. 7*A*, cell-free assay). The results (Fig. 7), including densitometry analysis, demonstrate that both full-length Efb and Efb-N disrupt the interaction between P-selectin and its endogenous ligand PSGL-1. (Fig. 7*A*). This was confirmed using platelet lysates as a source of native P-selectin and PSGL-1 (Fig. 7, *B* and *C*).

**FIGURE 8.**
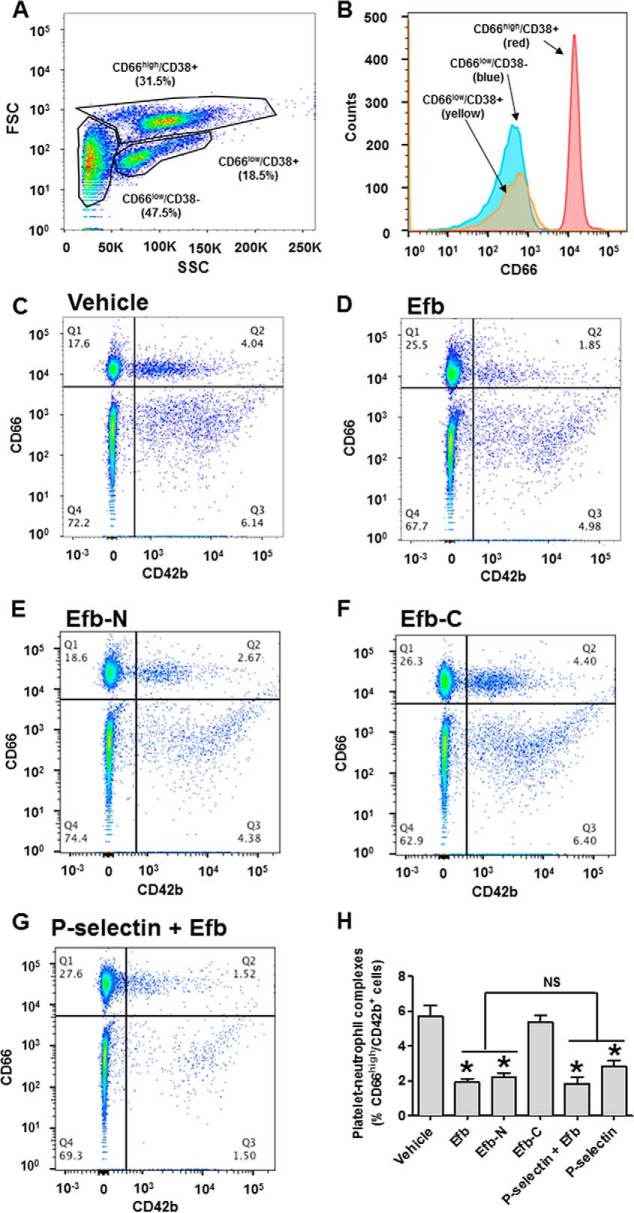
**Efb and Efb-N inhibit formation of platelet-granulocyte complexes.** Leukocytes from human blood were analyzed by flow cytometry (FACSAria III). *A*, forward scattering (*FSC*)/side scattering (*SSC*) dot plot identifies three distinct subpopulations. These populations were characterized as CD66^high^ cells (>5000 rfu in our experimental conditions), corresponding to granulocytes, and CD66^low^ cells, further divisible into CD38^+^ and CD38^−^. *B*, distribution of CD66 expression in the three subpopulations of leukocytes. Dot plots for the co-staining for CD66 (granulocytes) and CD42b (platelets) for untreated, 5 μm Efb-treated, 5 μm Efb-N-treated, or 5 μm Efb-C-treated whole blood are shown in *C*, *D*, *E*, and *F*, respectively. *G*, CD66/CD42b histogram for whole blood treated with both 50 μg/ml P-selectin-Fc and 5 μm Efb. In quadrant Q2 of the dot plots, the percentage of events with CD66 staining >5000 rfu and CD42b staining >500 rfu is represented. *H*, the portion of leukocytes positive for CD66 and CD42b staining (*i.e.* granulocyte-platelet complexes) for all tested conditions. Mean fluorescence value ± S.E. from four independent experiments is reported. Statistical significance was tested by one-way ANOVA with Bonferroni post test (* = *p* < 0.01 compared with vehicle, *NS* = non-significant in the pairwise comparison between Efb or Efb-N alone and treated with P-selectin or P-selectin and Efb, *n* = 4).

We next assessed whether Efb inhibition of P-selectin binding activity had any effect on the formation of platelet-leukocyte complexes that are induced by trans-cellular interaction of P-selectin and PSGL-1 (22, 23). As described previously (37), we investigated basal levels of platelet-leukocyte complexes in human whole blood by flow cytometry without additional *in vitro* platelet stimulation. CD42b (also known as glycoprotein 1b) was utilized as a specific marker for platelets. The leukocyte population comprised CD66^high^ cells (>5000 rfu in our experimental conditions), corresponding to granulocytes, and CD66^low^ cells (Fig. 8, *A* and *B*). In these experiments, blood particles characterized by high levels of CD66 and CD42b markers are complexes between granulocytes and platelets (Fig. 8*C*). Remarkably, blood incubation with 5 μm Efb or Efb-N significantly reduced the presence of granulocyte-platelet complexes, whereas 5 μm Efb-C did not affect granulocyte-platelet complex concentration (Fig. 8, *D--F*). In addition, we found that the addition of 50 μg/ml P-selectin-Fc significantly reduced platelet-granulocyte complexes when added alone (data not shown) but did not have any additive effect when added with Efb (Fig. 8*G*). Quantification of these results is shown in Fig. 8*H*.

**FIGURE 9.**
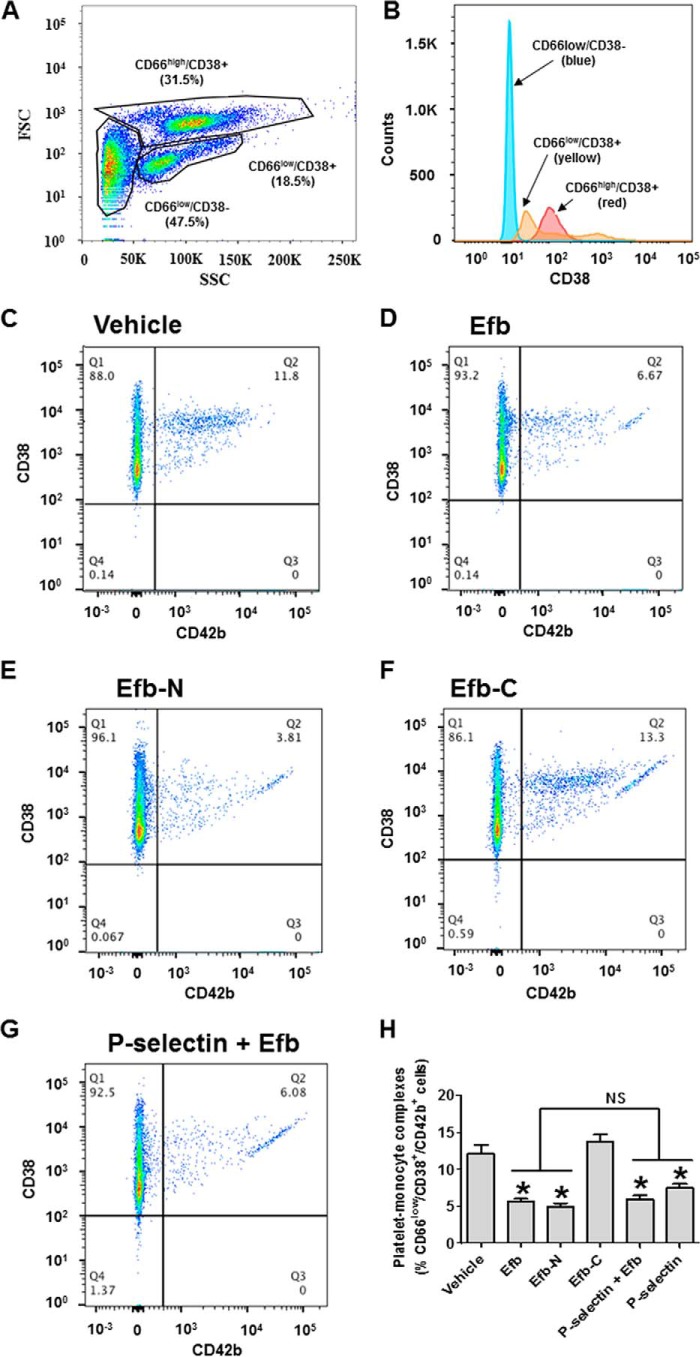
**Efb and Efb-N inhibit formation of platelet-monocyte complexes.** Leukocytes from human blood were analyzed by flow cytometry (FACSAria III). *A*, forward scattering (*FSC*)/side scattering (*SSC*) dot plot identifies three distinct subpopulations. These populations were characterized as CD66^high^ cells (>5000 rfu in our experimental conditions), corresponding to granulocytes, and CD66^low^ cells, further divisible into CD38^+^ and CD38^−^ (*i.e.* monocytes and lymphocytes). *B*, distribution of CD38 expression in the three subpopulations of leukocytes. Within the CD66^low^/CD38^+^ population, dot plots for the co-staining for CD38^+^ (granulocytes) and CD42b (platelets) for untreated, 5 μm Efb-treated, 5 μm Efb-N-treated, or 5 μm Efb-C-treated whole blood are shown in *C*, *D*, *E*, and *F*, respectively. *G*, CD38/CD42b histogram for CD66^low^/CD38^+^ population following treatment with both 50 μg/ml P-selectin-Fc and 5 μm Efb. In quadrant Q2 of the dot plots, the percentage of events with CD38 staining >100 rfu and CD42b staining >500 rfu is represented. *H*, the portion of CD66^low^/CD38^+^ leukocytes positive for CD42b staining (*i.e.* monocyte-platelet complexes) for all tested conditions. Mean fluorescence value ± S.E. from four independent experiments is reported. Statistical significance was tested by one-way ANOVA with Bonferroni post test (* = *p* < 0.01 compared with vehicle, *NS* = non-significant in the pairwise comparison between Efb or Efb-N alone and treated with P-selectin, or P-selectin and Efb, *n* = 4).

Non-granulocyte leukocytes (CD66^low^) are further divisible into lymphocytes and monocytes. Lymphocytes and monocytes were distinguished using CD38 as a monocyte marker (*i.e.* CD66^low^/CD38^+^ cells are monocytes, whereas CD66^low^/CD38^−^ cells are lymphocytes) (Fig. 9, *A* and *B*). We focused our attention on monocytes (CD38^+^). Blood particles positive for CD42b and CD38 within this population (CD66^low^/CD38^+^/CD42b^+^) are complexes between monocytes and platelets (Fig. 9*C*); these complexes were also decreased by incubation with 5 μm Efb or Efb-N (Fig. 9, *D* and *E*), but not Efb-C (Fig. 9*F*). Also, 50 μg/ml P-selectin-Fc significantly reduced platelet-monocyte complexes when added alone (data not shown) but did not have any additive effect when added with Efb (Fig. 9*G*). Quantification of these results is shown in Fig. 9*H*.

## Discussion

Similar to other proteins produced and released by *S. aureus*, including coagulase (Coa), extracellular matrix-binding protein (Emp), extracellular adhesive protein (Eap), staphylococcal complement inhibitor (SCIN), Sbi, and chemotaxis inhibitory protein (CHIPS) (3–5), Efb has attracted attention due to its role in the progression of *S. aureus* infection. Previous studies have highlighted the ability of Efb to inhibit platelet activation and hemostasis (18, 19). In addition, Efb has been shown to facilitate the escape of *S. aureus* from phagocytosis (15), exacerbate *S. aureus* infections (16, 20), and impair wound healing (17). The immunosuppressant role of Efb has been linked to its ability to interfere with the complement system and reduce bacterial phagocytosis (13, 15). The antihemostatic activity of Efb, however, has been suggested to depend on inhibition of fibrinogen binding by platelets or inhibition of platelet activation in response to fibrinogen binding, although the mechanism has not been resolved (18, 19). We therefore investigated the nature of Efb-platelet interaction at the molecular level.

In our thrombus formation and aggregation experiments, we localized the antiplatelet activity of Efb to its N-terminal domain, Efb-N (Fig. 1). This is in accordance with our binding experiments showing that full-length Efb and Efb-N bind the surface of platelets in the presence of thrombin or fibrinogen, whereas Efb-C does not (Fig. 3). This is also in accordance with previous studies showing antithrombotic activity of Efb N-terminal domain constructs (19). Our observations of Efb promotion of FITC-fibrinogen binding to platelets (Fig. 2), although initially unexpected, are in agreement with previous studies showing the promotion rather than inhibition of fibrinogen binding by platelets in the presence of Efb (18, 21); interestingly, these studies concluded that “enhanced Efb-dependent fibrinogen binding to platelets is of a nature that does not promote aggregation of the platelets” (*i.e.* “a non-functional interaction between platelets and fibrinogen”).

Our experiments with or without added extracellular fibrinogen have clarified the nature of Efb-platelet interaction and the role of fibrinogen, showing that there are two independent Efb-platelet binding modes: a fibrinogen-dependent binding mode mediated by integrin α_IIb_β_3_ (Fig. 4*D*) (which occurs in the presence of high extracellular concentrations of fibrinogen), and a novel platelet activation-dependent binding mode mediated by P-selectin but independent of fibrinogen (Fig. 6*B*) (which occurs in the absence of high extracellular concentrations of fibrinogen). This conclusion was reached in experiments on washed platelets; previous studies highlighted that the concentration of fibrinogen (<100 μg/ml) released by washed platelets *in vitro* (34, 35) is at least an order of magnitude lower than the average plasma concentration of fibrinogen in healthy blood donors of ∼3 mg/ml (33). Interestingly, several platelet functional responses depend on high concentrations of fibrinogen that cannot be attained by simple release by platelets. For example, platelet aggregation in response to weak agonists such as serotonin or ADP can take place only in PRP (where high fibrinogen concentrations are found) or in washed platelets with added fibrinogen or plasma proteins (38). It is therefore reasonable to assume that Efb binding to washed platelets in the absence of exogenous fibrinogen is fibrinogen-independent. This was confirmed by experiments using an inhibitory antibody for the main fibrinogen receptor on platelets, integrin α_IIb_β_3_, which showed no effect on Efb binding by platelets in the absence of exogenous fibrinogen. On the other hand, we proved that fibrinogen-dependent binding of Efb in the absence of stimulation is caused by basal levels of platelet and integrin α_IIb_β_3_ activation (Fig. 4, *E* and *F*).

The fibrinogen-independent binding of Efb to platelets is mediated by a platelet surface/extracellular protein that was previously unknown as a binding partner of Efb. Notably, the existence of an unidentified surface receptor for Efb was suggested by previous studies (18). We have now identified P-selectin as the surface protein responsible for mediating the interaction between Efb and platelets in the absence of high extracellular fibrinogen concentration. We initially identified P-selectin among platelet proteins as an Efb interactor using a proteomics approach (Table 1). We then confirmed this interaction using classical biochemical techniques (pulldown and immunoblot) and SPR. We showed that Efb-P-selectin interaction is fibrinogen-independent and occurs via Efb-N (Fig. 5). The translocation of platelet P-selectin to the platelet surface in response to platelet activation has been described (36) and explains why P-selectin-mediated binding of Efb becomes significant only after platelet activation (induced by thrombin in our experiments). The capability of Efb-N to bind both fibrinogen and P-selectin could be related to the intrinsically disordered nature of Efb-N (data not shown), noting that disorder often confers an increased repertoire of possible interactions on a protein domain (39).

The interaction between P-selectin and its endogenous ligand PSGL-1 is central to the formation of platelet-leukocyte complexes (40, 41) and the immune function of platelets (42). We therefore investigated the ability of Efb to affect the levels of heterotypic cell complexes between platelets and leukocytes in human blood. Platelet-granulocyte and platelet-monocyte complex levels were indeed significantly decreased by Efb and Efb-N (Figs. 8 and 9). In view of the role of platelet-leukocyte complex formation in the innate immune response (43), our findings suggest that at least part of the immunosuppressive role of Efb (16, 20) involves inhibition of platelet-leukocyte complex formation. In this respect, besides offering potential new angles to tackle *S. aureus* infections, understanding of the mechanism of action of Efb may have wider consequences. Platelet-leukocyte complexes have been shown to play an important role in inflammation (27, 44). As well as being useful research tools for the study of platelet-leukocyte complexes, peptides derived from Efb-N could therefore be the basis of a therapeutic agent for inflammatory diseases that are characterized by increased formation of platelet-leukocyte complexes; such diseases include atherosclerosis (45), autoimmune diseases (46, 47), and inflammatory thrombosis (48, 49). The interaction between P-selectin and its physiological ligand PSGL-1 has also been shown to play a critical role in the interaction of platelets with endothelial cells and the initiation of thrombosis (50–52). Therefore, this study may offer valuable information for the investigation or treatment of pro-thrombotic states induced by vascular inflammation and increased endothelial cell-platelet adhesion.

In summary, we have demonstrated for the first time a novel biological activity of the *S. aureus* protein Efb: inhibition of P-selectin-PSGL-1 interaction and consequent impairment of platelet-leukocyte complex formation. This function helps to explain the pathogenic role of Efb during *S. aureus* infection. Efb-based molecules may therefore be used to target the inflammatory function of platelets, which depends on P-selectin-dependent interaction with leukocytes.

## Author Contributions

M. G. P. and A. U. generated the constructs for the expression of Efb-C and did protein expression, purification, and labelling. M. G. P., A. U., S. B., and G. P. designed proteomics experiments. A. A. A. and I. C. performed immunoprecipitation and pulldown experiments. T. F. and D. V. performed flow cytometry experiments. S. B. initiated the project, generated constructs for the expression of Efb and Efb-N, provided expertise and support on protein chemistry, and co-wrote the manuscript. G. P. designed and analyzed platelet experiments, and co-wrote the manuscript. All authors were involved in data analysis.

## Supplementary Material

Supplemental Data

## Footnotes

3 The abbreviations used are:

Efb: extracellular fibrinogen-binding protein
Efb-C: C-terminal domain of Efb comprising residues 105–165
Efb-N: N-terminal domain of Efb comprising residues 30–104
ANOVA: analysis of variance
FGN: fibrinogen
PRP: platelet-rich plasma
PSGL-1: P-selectin glycoprotein ligand-1
rfu: relative fluorescence units.
