# Supplementary material for: Extracellular Fibrinogen-binding Protein (Efb) from Staphylococcus aureus Inhibits the Formation of Platelet-Leukocyte Complexes
Source: J Biol Chem. 2015 Dec 1;291(6):2764–76. doi: 10.1074/jbc.M115.678359 (PMC4742742; doi:10.1074/jbc.M115.678359)
Supplement: Supplemental Data [file supp_291_6_2764__index.html]

Extracellular Fibrinogen Binding Protein (Efb) from Staphylococcus aureus Inhibits the Formation of Platelet-Leukocyte Complexes — Extracellular Fibrinogen-binding Protein (Efb) from Staphylococcus aureus Inhibits the Formation of Platelet-Leukocyte Complexes — Efb Blocks Interaction between P-selectin and PSGL-1 — Supplemental Data 

# Extracellular Fibrinogen-binding Protein (Efb) from *Staphylococcus aureus* Inhibits the Formation of Platelet-Leukocyte Complexes

## Supplemental Data

- Supplemental Table 1 (.xlsx, 27 KB) - Proteomics data for identification of Efb binding proteins
